# Exogenous Plant-Based Nutraceutical Supplementation and Peripheral Cell Mononuclear DNA Damage Following High Intensity Exercise

**DOI:** 10.3390/antiox7050070

**Published:** 2018-05-21

**Authors:** Josh Williamson, Ciara M. Hughes, Gareth W. Davison

**Affiliations:** 1 Sport and Exercise Research Institute, Ulster University, Newtownabbey BT37 0QB, Northern Ireland; Williamson-J5@ulster.ac.uk; 2 Nursing and Health Research Institute, Ulster University, Newtownabbey BT37 0QB, Northern Ireland; cm.hughes@ulster.ac.uk

**Keywords:** reactive oxygen species, nutritional supplement, comet assay, oxidative stress

## Abstract

Plant-based nutraceuticals are categorised as nutritional supplements which contain a high concentration of antioxidants with the intention of minimising the deleterious effect of an oxidative insult. The primary aim of this novel study was to determine the effect of exogenous barley-wheat grass juice (BWJ) on indices of exercise-induced oxidative stress. Ten (*n* = 10) apparently healthy, recreationally trained (*V̇O_2max_* 55.9 ± 6 mL·kg^−1^·min^−1^), males (age 22 ± 2 years, height 181 ± 6 cm, weight 87 ± 8 kg, body mass index (BMI) 27 ± 1) volunteered to participant in the study. In a randomised, double-blinded, placebo-controlled crossover design, participants consumed either a placebo, a low dose (70 mL per day) of BWJ, or a high dose (140 mL per day) of BWJ for 7-days. Experimental exercise consisted of a standard maximal oxygen uptake test until volitional fatigue. DNA damage, as assessed by the single cell gel electrophoresis comet assay, increased following high intensity exercise across all groups (time × group; *p* < 0.05, Effect Size (ES) = 0.7), although there was no selective difference for intervention (*p* > 0.05). There was a main effect for time in lipid hydroperoxide concentration (pooled-group data, pre- vs. post-exercise, *p* < 0.05, ES = 0.2) demonstrating that exercise increased lipid peroxidation. Superoxide dismutase activity (SOD) increased by 44.7% following BWJ supplementation (pooled group data, pre- vs. post). The ascorbyl free radical (*p* < 0.05, ES = 0.26), α-tocopherol (*p* = 0.007, ES = 0.2), and xanthophyll (*p* = 0.000, ES = 0.5), increased between the pre- and post-exercise time points indicating a main effect of time. This study illustrates that a 7-day supplementation period of a novel plant-derived nutraceutical product is insufficient at attenuating exercise-induced oxidative damage. It is possible that with a larger sample size, and longer supplementation period, this novel plant-based nutraceutical could potentially offer effective prophylaxis against exercise-induced oxidative stress; as such, this justifies the need for further research.

## 1. Introduction

The physiological generation of reactive oxygen species (ROS) is an integral part of the biological redox equilibrium, and they play a salient regulatory role in cell signalling [1]. When the accumulation of ROS exceeds the endogenous antioxidant defence system, they incite an oxidative insult to important biological molecules, such as nucleic acids and lipids [2]. Consequently, ROS have been implicated in pathological diseases, including cancer and diabetes [3]. Moreover, the chronic, detrimental accumulation of ROS activates stress-sensitive intracellular pathways that generate downstream epigenetic modifications, resulting in cell damage and apoptosis [4].

Exercise-associated sources of ROS include mitochondrial oxidative leakage, NADPH oxidase and inflammatory processes [5,6], and there is a plethora of data demonstrating that exercise exacerbates the accumulation of ROS [7,8]. Consequently, this alters the redox balance towards a more pro-oxidant state, and in essence, oxidative stress must be controlled. Fortunately, the body possesses an elaborate endogenous defence system comprised of enzymatic and non-enzymatic antioxidants; including superoxide dismutase (SOD), catalase (CAT), ascorbic acid, and α-tocopherol [9]. These antioxidants contribute to the protection of redox disturbances within the cell, alongside preventing against ROS toxicity [10].

Additionally, there are numerous dietary antioxidants that can be consumed which contribute to an enhanced cellular protection. Ascorbic acid for example, effectively scavenges ROS and resynthesises α-tocopherol [11]. Equally, during lipid peroxidation, α-tocopherol acts as a reducing agent to peroxyl radicals; thus, inhibiting further propagation [12]. Indeed, there is extensive research demonstrating that antioxidant supplementation attenuates exercise-induced oxidative stress [13,14]. More recently, phytochemicals from plant-derived nutraceuticals have been utilised in animal studies investigating oxidative damage and the aging process [15], and Sweazea et al. [4] has shown a significant elevation in plasma catalase concentration, following oral administration of a plant-based nutraceutical.

Barley (*Hordeum vulgare* L.) and wheat grass (*Triticum aestivum*) are relatively novel oral plant-based nutraceuticals, however, research regarding their efficacy is lacking, particularly in the context of exercise and oxidative stress. The predominant components of barley grass are SOD, CAT, ascorbic acid, α-tocopherol, and glycosylisovitexin [16,17]. Ghavami and colleagues [18], illustrated that oral barley grass supplementation reduced radiation-induced DNA damage as measured by the comet assay. Similarly, wheatgrass contains an abundant source of ascorbic acid, α-tocopherol, and SOD which can attenuate the accumulation of ROS [19]. To elaborate, Sethi et al. [20] observed a reduction in lipid peroxidation, and a restoration of enzymatic antioxidant concentration following wheatgrass supplementation. To the best of our knowledge, no study has quantified the efficacy of supplementing with a novel wheat-barley grass juice blend on oxidative damage following exhaustive exercise. We therefore hypothesize, that supplementation with this unique plant-based nutraceutical will provide effective prophylaxis against exercise-induced oxidative stress. Thus, the primary aim of this study was to determine the efficacy of wheat and barley grass ingestion against intracellular DNA damage and lipid peroxidation.

## 2. Materials and Methods

### 2.1. Participants

Participants were recreationally active males, and all completed a medical history questionnaire, along providing written informed consent, prior to commencing the study. All participants were non-smokers, and free from any form of medication or antioxidant supplementation for 4 weeks prior to, and throughout the study. All participants were fasted for 12-h before experimental testing as this was necessary to standardize inter-participant blood biochemistry [21]. Participants could drink water *ad libitum*. The study was conducted in accordance with the Declaration of Helsinki and approved by a local University Ethics Committee (REC/16/0004).

### 2.2. Exercise Habituation

Prior to experimental testing, participants were instructed to attend the laboratory for preliminary baseline and anthropometric testing (Table 1). Following this, participants exercised for 10 min at 50% of maximum heart rate on a motorised treadmill (HP Comos, Hamburg, Germany) for the sole purpose of familiarisation.

### 2.3. Supplementation Protocol and Nutraceutical Preparation

**Figure 1:**
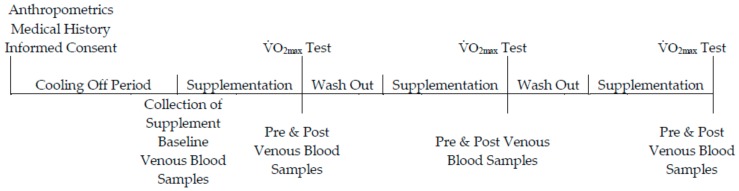
Schematic overview of the experimental protocol. Participants were allocated to one of the supplemental groups for 7 days. Following a 7-day washout period, participants crossed to another supplemental phase; this was repeated so each participant experienced each supplemented group. Participants completed a V̇O_2max_ (maximum oxygen uptake) test a total of 3 times throughout the study duration.

Using a randomised, double-blinded, placebo-controlled, crossover design, participants were allocated to one of three groups. Participants allocated to the placebo (*n* = 10) group consumed 70 mL per day of a liquid-form placebo. Participants assigned to the low-dose (*n* = 10) and high-dose (*n =* 10) group consumed 70 mL or 140 mL per day of a fresh plant-based nutraceutical (BWJ: Tiro Nutrition; Northern Ireland, UK), respectively. The supplementation phase lasted 7-days, followed by a 7-day wash-out period before commencing the next phase of supplementation [22]. An overview of the experimental design is outlined in Figure 1. The fresh barley-wheat grass juice (BWJ) was prepared as follows: the young shoots of barley (*Hordeum vulgare* L.) and wheat (*Triticum aestivum*) grass were grown indoors and a single true leaf was harvested 10–14 days following gemination; leaves were cold-pressed to extract the juice using a slow speed twin auger screw press. A juice-blend of 50:50 from barley-wheat grass was combined with 1–2% volume of lemon juice; the final product was bottled into polyethylene terephthalate bottles and treated by high pressure pasteurization.

### 2.4. Experimental Testing

Participants abstained from exercise and alcohol consumption for 48 h before completing a standardized maximal oxygen uptake test. To negate the possibility of a diurnal variation effect on blood indices, all participants attended the laboratory at the same time (08:00) on each experimental day. Briefly, a motorized treadmill was set at 11 km/h with a 1% gradient rise at each 1-min interval until volitional fatigue. Validation of V̇O_2max_ was confirmed when the respiratory exchange ratio was above 1.15 arbitrary units, a plateau in the oxygen uptake/exercise intensity relationship (>2 mL kg^−1^ min^−1^), and a heart rate within 10 beats min^−1^ of age-predicted maximum. All participants across each of the experimental phases achieved V̇O_2max_.

### 2.5. Biochemical Indices

Blood was extracted from a prominent antecubital forearm vein before supplementation, and then again following supplementation (pre-exercise) and post-exercise. All blood was centrifuged, aliquoted, and stored at −80 °C prior to biochemical analysis.

An exercise-induced hemoconcentration was determined using the equations of Dill and Costill [23], incorporating haemoglobin and haematocrit indices; this was used to account for acute-exercise induced plasma volume changes. Packed cell volume (%) was measured using the microcapillary reader technique, and corrected by 1.5% for plasma trapped within erythrocytes [24].

### 2.6. Deoxyribonucleic Acid (DNA)

DNA damage was measured in human peripheral blood mononuclear cells (PBMCs) using the comet assay as detailed by Singh et al. [25]. Briefly, the PBMCs were isolated by layering 3 mL of whole blood onto 3 mL of Histopaque-1077 (Sigma-Aldrich, St. Louis, MO, USA), and centrifuged at 3500 rpm for 30 min at 4 °C. Next, 50 µL of cells were mixed with 150 µL of Low Melting Point Agarose, of which 70 µL was layered on to prepared Normal Agarose slides and allowed to solidify under coverslips at 4 °C. After 5 min, the coverslips were removed and placed in lysis buffer (2.5 m NaCl, 100 mM NaEDTA, 10 mM Trizma, 1% Tition-X, pH 10), for 1 h at 4 °C. Slides were then placed in electrophoresis buffer (300 mM NaOH, 1 mM EDTA, pH 12.5–13) for a 20-min incubation period, followed by 30 min electrophoresis at 4 °C (25 V, 300 mA). Slides then underwent neutralisation followed by staining using SYBR^®^ Gold (Thermo Fisher Scientific, Waltham, MA, USA). 50 random cells were counted at magnification 400× utilizing an Olympus BH-2 epifluorescence microscope. All steps were carried out in the dark to prevent further DNA damage. The intra/inter-assay coefficient of variation (CV’s) was <8%.

### 2.7. Lipid Hydroperoxides (LOOH)

Serum LOOH was measured spectrophotometrically using the method of Wolff [26]. Briefly, ferrous oxidation of Xylenol Orange (FOX) was used to quantify the oxidation of ferrous (Fe^2+^) iron to ferric (Fe^3+^) iron ions, and the subsequent binding of Fe^3+^ to the FOX-1 reagent. The intra/inter-assay coefficient of variation (CV’s) was <5%.

### 2.8. Lipid Soluble Antioxidants (LSA)

LSA were analysed by simultaneous determination using the high-performance liquid chromatography (HPLC) method as described by Thurnham et al. [27]. The intra/inter-assay coefficient of variation (CV’s) was <7%.

### 2.9. Electron Paramagnetic Resonance (EPR) Spectroscopy

The ascorbyl free radical was measured using EPR on a Bruker EMX spectrometer (Bruker Instruments Inc., Billerica, MA, USA) as described previously by Clifford et al. [28]. Firstly, 1 mL of plasma was mixed thoroughly with 1 mL of dimethyl sulfoxide (DMSO) in a glass test tube, and 1 mL of the final solution was drawn into a sterile syringe and flushed into the cavity. All samples were analysed at room temperature. The spectrometer parameter conditions were set as follows: frequency (9.785 GHz); microwave power (20 mW); modulation frequency (100 kHz) and modulation amplitude (1.194 G) for three sweeps. Spectral parameters were obtained using commercially available software (Bruker Win EPR System, Version 3.2, Bruker Instruments Inc., Billerica, MA, USA) and filtered identically. The relative concentration of the ascorbyl free radical was determined by single spectra signal intensity.

### 2.10. Superoxide Dismutase (SOD)

Extracellular SOD activity was measured spectrophotometrically using a Superoxide Dismutase Assay Kit (Cayman Chemicals, Ann Arbor, MI, USA). SOD was quantified by utilising formazan dye via tetrazolium salts following the administration of xanthine oxidase to the serum samples. The intra-assay coefficient of variation was 3.2%.

### 2.11. Statistical Analysis

The method of Cohen was utilized to calculate the prospective power of the test based on DNA damage data published by Fogarty et al. [29]. SPSS statistical software (IBM, Surrey, UK, v.23) was used to analyse data sets, and data normality was determined using the Shapiro-Wilks test (*p* > 0.05). A two-way, repeated-measures ANOVA ascertained differences between groups and across time. Following a significant interaction effect (time × group, *p* < 0.05), between group differences were subsequently analysed using a one-way ANOVA, while a Bonferroni paired samples *t*-test was used for within time differences. All significant changes were established at *p* < 0.05. The magnitude of change is expressed as partial eta squared (effect size, ES) throughout.

## 3. Results

### 3.1. Compliance

Participant characteristic data and performance variables during each of the supplemental phases are presented in Table 1. All ten participants (100%) completed all three arms of the crossover trial. There was a 99% compliance of supplementation ingestion as one participant experienced emesis on two occasions following consumption of BWJ during the high-dose phase.

### 3.2. DNA Damage

**Figure 2:**
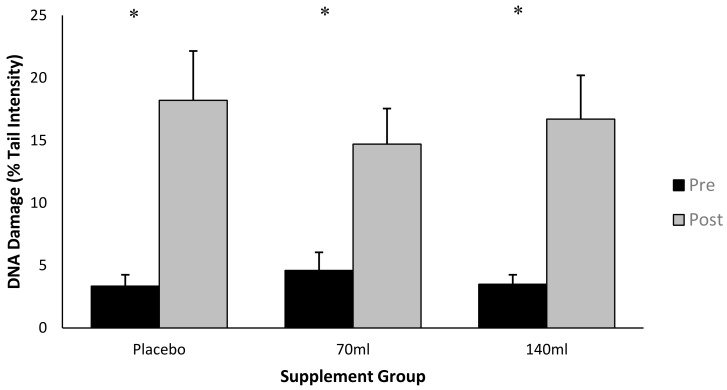
DNA damage expressed as Tail % intensity at pre- and post-exercise across groups (*n* = 10). Data expressed as a mean ± standard deviation.* represents a significant interaction effect (*p* < 0.05) within group.

Data is presented as % tail DNA, where an increase in % tail DNA demonstrates an increase in DNA damage (Figure 2). There was an interaction effect for time × group (*p* < 0.05, ES = 0.6), and the post-hoc analysis indicated a difference between pre- and post-exercise for each of the three groups (*p* < 0.05, ES = 0.7). There was also a main effect for time (pooled group pre- vs. post-exercise, *p* < 0.05, ES = 0.7). Collectively this data demonstrates that exercise increased DNA damage. The increase in DNA damage as a function of exercise *per se* was Δ (delta change is expressed as the percentage change from pre- to post-exercise) 18.2%, Δ9% and Δ13% for placebo, low-dose and high-dose barley grass groups respectively, suggesting that the barley grass partially attenuated the rise in DNA damage compared with the placebo; however, these changes were not significant (*p* > 0.05).

### 3.3. Lipid Hydroperoxide

**Figure 3:**
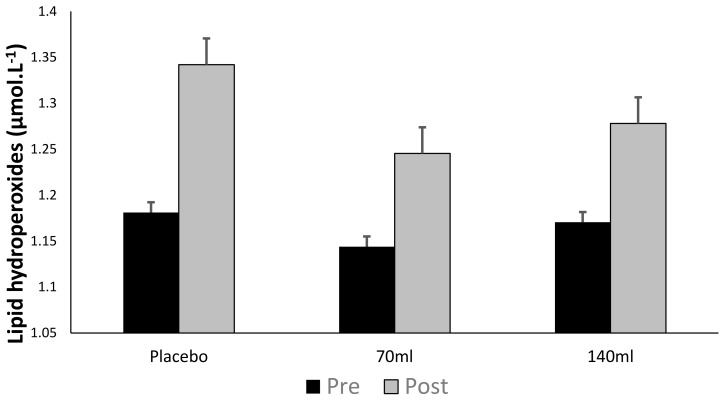
Lipid hydroperoxides (mean ± SD) at pre- and post-exercise across groups (*n* = 10).

There was no time × group interaction (*p* > 0.05) as observed in Figure 3; however, there was a main effect for time (pooled group pre- vs. post-exercise, *p* < 0.05, ES = 0.26), i.e., exercise increased lipid peroxidation. Within individual groups, lipid hydroperoxides increased by Δ12.6%, Δ7.3% and Δ7.8% for placebo, low- and high-dose barley grass groups respectively, suggesting that the BWJ supplementation partially attenuated the rise in lipid hydroperoxides compared with placebo (although this was not significant as a function of time × group).

### 3.4. Lipid Soluble Antioxidants

The recorded lipid soluble antioxidant (LSA) concentrations found in Table 2 demonstrate an interaction effect of time × group in γ-tocopherol (*p* = 0.03, ES = 0.03, Δ27.1%) within the placebo group; however, there was no significant interaction effect between supplemental groups and time on any of the other LSA, (*p* > 0.05). A main effect of time (pooled data) showed an increase (*p* < 0.05) between baseline and post-exercise time points for α-tocopherol (Δ10.5%), γ-tocopherol (Δ23.5%), and xanthophyll (Δ64.2%) concentrations. The main effect of time (pooled date) also demonstrated an increase in α-tocopherol (*p* = 0.007, ES = 0.2, Δ7.9%) and xanthophyll (*p* = 0.000, ES = 0.51, Δ14.9%) between pre- and post-exercise.

### 3.5. Ascorbyl Free Radicals

**Figure 4:**
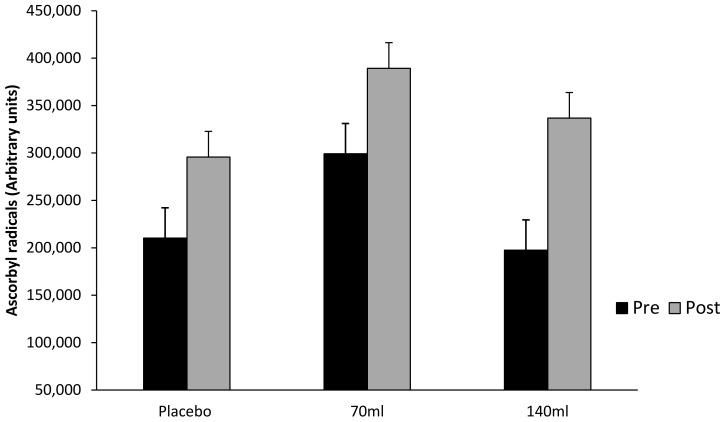
Ascorbyl free radical concentration (mean ± SD) at pre- and post-exercise across groups (*n* = 10).

Figure 4 demonstrates no interaction effect either within or between groups (*p* > 0.05), however, there was a main effect for time (pooled group pre- vs. post-exercise, *p* < 0.05, ES = 0.26, Δ44.5%) demonstrating that exercise increased ascorbyl free radical concentration.

### 3.6. Superoxide Dismutase

**Figure 5:**
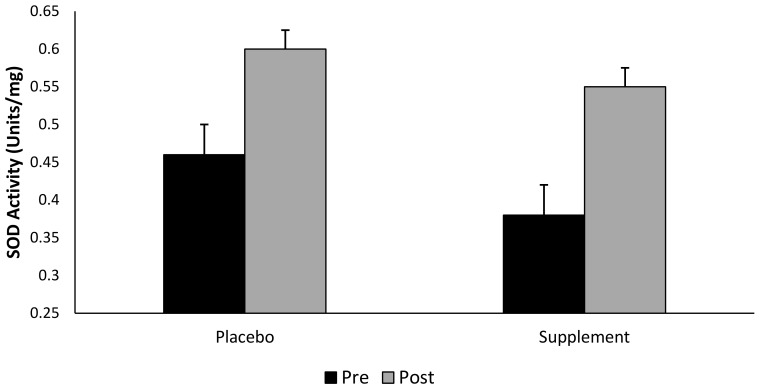
Superoxide dismutase activity (SOD) (mean ± SD) at pre- and post-exercise across groups (*n* = 10). Time points comparing placebo to pooled barley grass groups. Values expressed as mean and standard deviation.

Although there was no interaction effect for group × time (*p* > 0.05), SOD increased by Δ 44.7% in the supplemented group (pooled supplement data), and Δ30% in the placebo group following a single bout of exhaustive exercise (Figure 5). That said, there was a main effect observed for both group and time (pooled data, *p* < 0.05).

## 4. Discussion

The primary aim of this study was to determine the antioxidant effect of a novel plant-based nutraceutical supplement on exercise-induced oxidative damage. This study demonstrates that exhaustive, high-intensity exercise leads to DNA damage and lipid peroxidation, and it is conceivable these perturbations may disrupt normal biological function. There was no clear antioxidant effect with regards to selectively attenuating DNA and lipid damage following exercise. That said, the novel BWJ supplement as observed, and expressed as a delta change, seems to marginally attenuate oxidative damage (although not from a statistical perspective), and further work is merited to fully explore this relationship.

### 4.1. DNA Damage

Previous research has established that exhaustive exercise is sufficient to induce oxidative modification to DNA and lipids [13,30]. The present study corroborates this supposition by demonstrating that DNA damage as measured by the comet assay, increases following maximal exercise (*p* < 0.05, ES = 0.73, Δ13.4%). The comet assay is a reliable and sensitive technique for detecting DNA strand breaks [31], and has been utilised within exercise-related research [32].

ROS associated with exercise, are generated through several sources including mitochondrial oxidative phosphorylation, and enzymatic complexes such as NADPH oxidase and xanthine oxidase [33]. To elaborate, the literature demonstrates that superoxide is produced from NADH dehydrogenase and cytochrome c oxidoreductase of the electron transport chain [34]. Mitochondrial ROS generation is a by-product of normal metabolism and as a result, the persistent nature of oxidative phosphorylation renders mitochondria one of the primary sources of endogenous ROS [35]. It should be noted that this mechanism is somewhat controversial, and research suggests that ROS generation from the electron transport chain is greater at rest when compared to exercise [36,37]. As suggested by Austin et al. [38], it is also possible that during exercise, the increase in mitochondrial respiration causes an increase in hydrogen peroxide via peroxisome proliferator-activated receptor γ coactivator 1α (PGC-1α). Paradoxically, as mitochondrial activity increases, PGC-1α regulates the expression of enzymatic antioxidants such as SOD and CAT [39]. As a final point, it is highly probably that the observed increase in oxidative DNA damage, is a partial consequence of myofibril damage from the exhaustive exercise. In turn, the activation of phagocytes, and potentially endothelial xanthine oxidase, may enhance the production of ROS; resulting in peripheral leukocyte damage [40]. Excessive concentrations of superoxide can promote the formation of other ROS which directly damage DNA and lipids [41].

The data from our study, which indicates a single bout of exercise is sufficient to induce DNA damage, concurs in line with previously published literature [13,14,42]. We propose that this exercise-induced DNA modification (albeit single stranded DNA damage) may perhaps be beneficial for downstream cell signalling. It is conventionally accepted that the chronic accumulation of ROS is implicated in pathological conditions as a consequence of oxidatively modified DNA base lesions [43]. Specifically, the lesion 7,8,-dihydro-8-oxoguanine (8-oxoG), and subsequent product 2,6-diamino-4-hydroxy-5-formamido-pyrimidine, is most abundantly generated due its low redox potential [44]. To elaborate, baseline levels of 8oxo-G are significantly elevated in cancer patients, when compared to healthy individuals [45]. The removal of these lesions is primarily repaired through the base excision repair (BER) pathway, via 8-oxoguanine DNA glycosylase1 (OGG1) [46]. Recent evidence demonstrates that the knockout of OGG1 leads to the supra-physiological accumulation of 8-oxoG; subsequently leading to anomalous immune responses and metabolic disorders [47]. Additionally, activation of OGG1 causes the downstream expression of specific transcription factors as a function of an oxidative insult [48]. Recently, Radak and colleagues [47], have elucidated the complex relationship between 8-oxoG, OGG1, and the NF-κB-driven gene-expression pathway. Although the mechanisms are not currently well understood, it is clear that DNA base lesions, and their cognate repair enzymes, play critical roles in signal-transduction pathways. This highlights the inherent need to investigate whether antioxidant supplementation is beneficial to these repair pathways, or if they hinder signal-transduction regulation and downstream repair processes. We are mindful however, that the DNA changes quantified, and observed in our experiment is single-stranded damage, and not base oxidation *per se*, and future work should focus on DNA-BER repair pathways and mechanisms, to provide context to the notion that BER is activated following exercise-induced DNA oxidation.

### 4.2. Lipid Peroxidation

From our data, lipid hydroperoxides increased following exercise (Δ9.2%), but there was no selective difference as a function of supplementation. Previously published data demonstrates that exercise can exacerbate lipid hydroperoxides [49] which can subsequently damage DNA [50].

Lipid hydroperoxides *per se* are by-products of the lipid peroxidation cascade pathway, and they form via hydrogen abstraction from a polyunsaturated fatty acid side chain [7,51]. Oxidation of vascular lipid-membranes can generate lipid hydroperoxides through the exercise-induced production of molecular free radical species [14]. This decomposition of polyunsaturated fatty acids can produce an array of mutagenic compounds, and damaging intermediates, including alkoxyl free radicals [52] and malondialdehyde [49]; both molecules are capable of directly damaging DNA, whilst alkoxyl free radicals may perpetuate lipid peroxidation [52].

### 4.3. Prophylactic Effect of Plant-Derived Nutraceuticals

Research ascertains that barley and wheat-grass contain enzymatic antioxidants such as SOD [53,54], glutathione peroxidase (GPx), and CAT [55]. Previous work has also identified significant concentrations of ascorbic acid and α-tocopherol within young shoots of barley and wheat-grass [17,48]. Moreover, the identification of these antioxidants, and following barley grass supplementation, oxidative stress is reduced in type 2 diabetes mellitus [56].

Although a marginal attenuation in DNA damage and lipid peroxidation following supplementation was observed, there was no significant interaction effect between supplementation and any parameter of exercise-induced oxidative stress. Given the novel nature of the nutraceutical, it is difficult to ascertain the specific active antioxidants without the use of ultra-high-performance lipid chromatography-mass spectrometry (UHPLC-MS). While this study has provided pioneering data to the body of literature, quantification of enzymatic and non-enzymatic antioxidants via UHPLC-MS would offer invaluable guidance for supplementation dose and length of the supplementation period. Previous research by Yi et al. [57], and Ben-Arye et al. [58], demonstrated a significant interaction effect by supplementing with 100 mL of wheat grass for 14 days, and 30 days, respectively. It should be noted however, that both these studies have different sample populations, study designs, and biochemical parameters of oxidative stress, compared to the present study. Although our nutraceutical is a combined barley-wheat grass blend, the presented literature, and data from our study, suggests future research groups should consider a minimum of 14 days supplementation and/or a higher supplementation dose.

Due to ROS scavenging capabilities, an activated reduction in the concentration of LSA may coincide with a reduction in the biomarkers associated with an oxidative insult [59]. Both Traber & Atkinson [60] and McAnulty et al. [61], suggest that α-tocopherol concentration can decline within the vascular circulation as a function of acute exercise. Contrary to this, antioxidant supplementation can increase the systemic concentration of lipid- and aqueous-soluble antioxidants [62,63]. Although not statistically significant, many of the LSA increased following exhaustive exercise, perhaps suggesting an activation of lipolysis, and subsequent antioxidant releasing effect into the circulation [64]. In agreement, both Long et al. [65] and Fogarty et al. [59], suggest this can be exacerbated when exercising in a fasted state. Supplementation of BWJ increased xanthophyll concentration only (Δ69.7%), and work has shown that xanthophyll is an effective antioxidant with regards protecting against DNA damage and lipid peroxidation [66]. Although participants were under clear instruction to follow their habitual dietary intake, it is important to note that we did not strictly control for diet *per se*, and it is indeed conceivable that a heterogeneous diet may influence the systemic concentration of LSA [67,68]. However, within the present study, there was no difference observed in blood lipid soluble antioxidant concentrations between groups at the pre-exercise time point; thus, supporting the notion that diet remained constant across the study. Additionally, the authors are confident that the randomized, crossover design of the study minimised any significant dietary variation.

Recent research demonstrates an increase in SOD, GPx and glutathione reductase activity following exhaustive exercise to volitional fatigue [69]. Additionally, Khassaf and colleagues [70], has shown an elevation in muscle cell SOD activity following a single bout of exhaustive exercise. The findings of the present study showed no significant interaction between intervention groups or across time points. Nevertheless, and whilst taking into consideration the pooled (pre- vs. post-exercise) time points, our data illustrates that supplementing with BWJ provides a Δ46% increase in SOD activity compared to a Δ30% increase in the placebo group. Considering the marginal reduction in DNA and lipid hydroperoxides damage following supplementation, alongside the elevation in extracellular SOD observed in the supplemented groups, it is theoretically plausible that BWJ provided a degree of cell protection against an exercise-induced oxidative insult; however, further research is warranted to confirm this supposition.

It should be noted that there are limitations with the current study, and any future experiment using this novel based nutraceutical should include more than double the existing sample size; this assumes that any similar investigative study involves using apparently healthy male volunteers of a similar age and body mass index as that used in our study. Although a prospective calculation of power was performed, calculating the power of the test retrospectively (power = 0.3) highlights the inherent weakness of performing such calculations prior to testing.

## 5. Conclusions

This investigation confirms previous work (from our laboratory and others) that exhaustive exercise can cause DNA and lipid damage, and that the novel BWJ nutraceutical product used does not statistically attenuate the observed cell damage. That said, our findings may be viewed as preliminary evidence that this plant-derived nutraceutical supplementation may have some potential beneficial effects with regards reducing an exercise-associated oxidative insult, and further work in this area should focus on a revised participant number and the length of supplementation period. Moreover, it is imperative to determine the exact mechanistic based properties; it is therefore necessary to perform UHPLC-MS on the specific plant-derived nutraceutical to provide a better understanding of the constituents within the specific product.

## Figures and Tables

**Table 1:** Participant characteristics (*n* = 10). All values are expressed as Mean ± Standard Deviation. Abbreviations: cm—centimetres; kg = kilograms; yrs—years; HR = heart rate; bpm = beats per minute, V̇O_2max_—maximum oxygen uptake; TTE = time to exhaustion; mins = minutes.

Participant Characteristics	Baseline Data		
Age (yrs)	21.5 ± 2		
Height (cm)	180.5 ± 6		
Weight (kg)	86.6 ± 8		
Resting HR (bpm)	69.5 ± 5		
**Exercise Characteristics**	**Placebo**	**Low**	**High**
Maximum HR (bpm)	189 ± 8	187 ± 8	189 ± 8
V̇O_2max_ (mL kg^−1^ min^−1^)	55.1 ± 6	57.2 ± 5	55.4 ± 7
TTE (mins)	9.3 ± 2	8.4 ± 2	8.5 ± 2

**Table 2:** Lipid soluble antioxidants at rest (pre-exercise) and following exhaustive exercise for placebo, low-dose and high-dose supplemented groups. All values are expressed as means ± standard deviation and expressed as mmol·L^−1^. B = Baseline; R = Rest; E = Exercise. * denotes significant interaction effects of group and time (*p* < 0.05). ^#^ denotes significant main effects of time at the pre vs. post exercise time points.

Lipid Soluble Antioxidants	Baseline	Rest	Exercise	Δ% (B−R)	Δ% (R−E)
*α-Tocopherol*					
Placebo	20.90 ± 1.7	21.17 ± 4.1	23.76 ± 4.3	1.3	12.2 ^#^
Low	20.90 ± 1.7	21.71 ± 3.5	22.97 ± 3.8	3.9	5.8 ^#^
High	20.90 ± 1.7	21.30 ± 3.8	22.53 ± 3.5	1.9	5.8 ^#^
*γ-Tocopherol*					
Placebo	1.19 ± 0.4	1.44 ± 0.1	1.83 ± 0.7	21.0	27.1 *
Low	1.19 ± 0.4	1.25 ± 0.4	1.17 ± 0.3	5.0	−6.4
High	1.19 ± 0.4	1.44 ± 0.5	1.41 ± 0.5	21.0	−2.1
*α-Carotene*					
Placebo	0.05 ± 0.01	0.05 ± 0.01	0.05 ± 0.01	0	0
Low	0.05 ± 0.01	0.04 ± 0.01	0.04 ± 0.01	−20.0	0
High	0.05 ± 0.01	0.05 ± 0.01	0.04 ± 0.01	0	−20.0
*Retinol*					
Placebo	1.96 ± 0.4	1.94 ± 0.5	2.20 ± 0.5	−1.0	13.4
Low	1.96 ± 0.4	1.94 ± 0.4	2.14 ± 0.6	−1.0	10.3
High	1.96 ± 0.4	2.00 ± 0.5	2.32 ± 0.3	2.0	16.0
*Xanthophyll*					
Placebo	0.28 ± 0.1	0.30 ± 0.1	0.37 ± 0.1	7.1	23.3 ^#^
Low	0.28 ± 0.1	0.50 ± 0.3	0.54 ± 0.3	78.6 ^#^	8.0 ^#^
High	0.28 ± 0.1	0.45 ± 0.1	0.51 ± 0.2	60.7 ^#^	13.3 ^#^
